# Driver mutation-defined molecular landscapes of essential thrombocythemia

**DOI:** 10.1097/BS9.0000000000000273

**Published:** 2026-01-14

**Authors:** Alejandra L. Lorenzen, Gang Huang

**Affiliations:** a Department of Cell Systems and Anatomy, UT Health San Antonio, Joe R. and Teresa Lozano Long School of Medicine, San Antonio, Texas, USA; b Department of Pathology and Laboratory Medicine, UT Health San Antonio, Joe R. and Teresa Lozano Long School of Medicine, San Antonio, Texas, USA; c MD Anderson Mays Cancer Center at UT Health San Antonio, San Antonio, Texas, USA

Essential thrombocythemia (ET), a Philadelphia-negative myeloproliferative neoplasm, is driven by mutations in Janus kinase 2 (JAK2), calreticulin (CALR), or myeloproliferative leukemia protein (MPL), though 12% of patients lack these canonical drivers, referred to as triple-negative ET (TN-ET).^1^ Despite their genetic heterogeneity, these mutations converge on similar clinical presentations of excessive platelet production and increased thrombotic risk but lead to different outcomes in transformation and prognosis.^1,2^ How specific driver mutations reshape hematopoietic stem cell (HSC) programs has remained unclear, limiting the development of mutation-informed therapies beyond the standard of care of interferon or hydroxyurea.^3,4^ In a recent issue of *Advanced Science*, Tong et al^5^ address this gap by performing single-cell RNA sequencing with parallel driver mutation detection (3’-TARGET-seq) on highly purified HSCs from treatment-naïve patients across the ET spectrum: JAK2 V617F (n = 7), CALR (n = 13), MPL (n = 10), and triple-negative (n = 5), compared to normal controls (n = 15). This comprehensive analysis reveals both mutation-specific signatures and shared features underlying ET pathogenesis.

**Figure 1.**
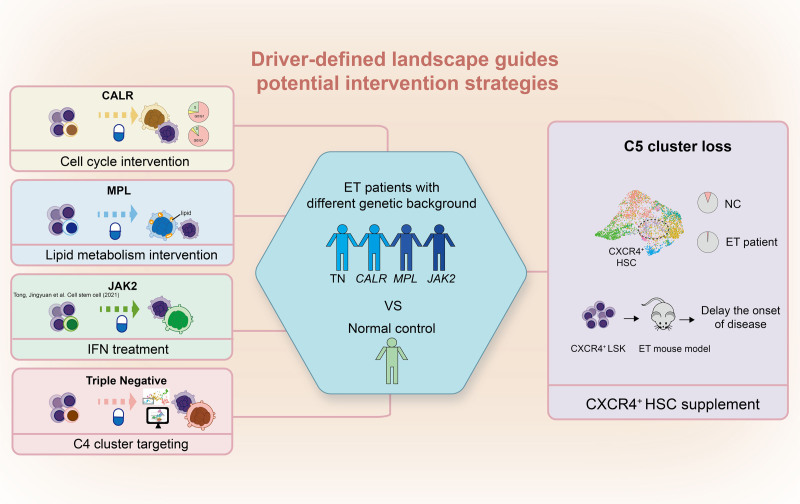
Driver-defined molecular landscapes of ET and their implications for tailored intervention strategies. Schematic summary illustrating how distinct mutation-specific alterations uncovered in HSCs—lipid metabolic rewiring in MPL-mutated ET, cell cycle-related pathway activation in CALR-mutated ET, and transcriptionally aberrant HSC states in TN-ET—highlight potential therapeutic vulnerabilities across ET subtypes. Meanwhile, a unifying concept of disrupted HSC population balance was also depicted, characterized by loss of CXCR4^+^ HSCs, providing a framework for strategies aimed at restoring stem cell homeostasis as a complementary therapeutic approach. CALR = calreticulin, ET = essential thrombocythemia, HSC = hematopoietic stem cell, IFN = interferon, MPL = myeloproliferative leukemia protein, NC = normal control, TN-ET = triple-negative ET.

The authors made 3 key contributions to understanding ET pathogenesis. First, they defined distinct molecular programs for each mutation. MPL-mutated HSCs exhibit aberrant cholesterol and lipid metabolism, characterized by elevated SREBF1/2 and FASN expression, which drives megakaryopoiesis through endoplasmic reticulum (ER) stress. CALR-mutated HSCs show hyperproliferation mediated by mechanistic target of rapamycin complex 1 (mTORC1) signaling, with type 2 mutations demonstrating even stronger proliferative activity than type 1 mutations—potentially explaining their higher platelet counts and better interferon response. In addition, they identified JAK2 V617F-mutated HSCs display enhanced inflammatory signatures alongside megakaryocyte priming. Second, the authors identified a consistent depletion of a CXCR4^+^ HSC subset (C5) across all ET subtypes. This lymphoid-biased population is enriched for “young HSC” signatures and chemokine genes. Its loss correlates with myeloid skewing, and transplantation experiments demonstrate that supplementing CXCR4^+^ cells delays MPN onset and reduces platelet counts in mice. Third, TN-ET harbors a proliferative HSC subset (C4) that transcriptionally resembles driver-mutated HSCs despite lacking canonical mutations, suggesting disease can arise from dysfunctional HSC states even in the absence of genetic drivers, potentially explaining why mutations can exist for years as clonal hematopoiesis before clinical manifestation (**Fig. 1**).

These observations shed intriguing and important insights into potential personalized and tailored therapeutic strategies of ET. For MPL-ET patients, lipid metabolic rewiring appears to interact with thrombopoietin/MPL signaling, driving aberrant megakaryopoiesis. Future mechanistic dissection and exploration of potential metabolic vulnerabilities in MPL-ET patients would be required. In CALR-ET patients, prominent activation of mTOR signaling was detected in *CALR*-mutated HSCs, and functional inhibition with rapamycin supports the idea that this pathway represents a mutation-clonal vulnerability. In TN-ET, these findings raise questions about the stability, functional behavior, and therapeutic susceptibility of aberrant HSC states, which may represent the true disease-initiating population in TN-ET.

However, there are considerable limitations that warrant caution and further investigation. The CXCR4^+^ findings present a conceptual contradiction: depleting CXCR4^+^ HSCs increases myeloid output, whereas genetic CXCR4 knockout studies show impaired myelopoiesis. This discrepancy likely reflects the difference between removing a subset vs completely abolishing CXCR4 function across all hematopoietic cells. Nonetheless, it remains unclear whether CXCR4 serves merely as a marker or is a functional requirement. From a therapeutic perspective, systemic inhibition of SREBF/FASN or mTOR risks impairing normal hematopoiesis, and supplementation with CXCR4^+^ HSC is currently clinically impractical. Additionally, the bone marrow microenvironment alterations inferred from non-mutated HSC require spatial transcriptomics and proteomics validations.

In summary, by revealing mutation-specific HSC programs alongside shared C5 depletion, this study advances ET pathogenesis understanding beyond clonal dominance toward HSC heterogeneity disruption. This systems-level perspective has far-reaching implications not only for ET biology but also for improving transplantation and therapeutic strategies, where restoring or preserving balanced HSC heterogeneity may be critical for normal hematopoiesis. Future studies should include longitudinal tracking from clonal hematopoiesis through ET onset, HSC-specific conditional CXCR4 deletion combined with fate-mapping, single-cell multi-omics for TN-ET, and mutation-stratified clinical trials incorporating serial HSC profiling. This pioneering work lays a solid foundation that warrants validation through mechanistic dissection and clinical correlation of the stem cell basis of ET pathogenesis before translating to precision therapies for ET.
